# Crystal structure of 2-[bis(benzylsulfanyl)methyl]-6-methoxyphenol

**DOI:** 10.1107/S2056989020002091

**Published:** 2020-03-03

**Authors:** Abhinav Raghuvanshi, Lena Knauer, Lydie Viau, Michael Knorr, Carsten Strohmann

**Affiliations:** a Institut UTINAM UMR 6213 CNRS, Université Bourgogne Franche-Comté, 16, Route de Gray, 25030 Besançon Cedex, France; b Anorganische Chemie, TU Dortmund University, Otto-Hahn-Str. 6/6a, D-44227 Dortmund, Germany

**Keywords:** crystal structure, *ortho*-vanillin, thio­acetal, di­thio­ether, supra­molecular network

## Abstract

The title compound, C_22_H_22_O_2_S_2_, **1**, represents an example of an *ortho*-vanillin-based functionalized di­thio­ether, which could be useful as a potential chelating ligand or bridging ligand for coordination chemistry. This di­thio­acetal **1** crystallizes in the ortho­rhom­bic space group *Pbca*. The phenyl rings of the benzyl groups and that of the vanillin unit form dihedral angles of 35.38 (6) and 79.77 (6)°, respectively. The crystal structure, recorded at 100 K, displays both weak intra­molecular O—H⋯O and inter­molecular O—H⋯S hydrogen bonding.

## Abstract 2

The title compound is an example of an *ortho*-vanillin-based functionalized di­thio­ether, which could be useful as a potential chelating ligand or bridging ligand for coordination chemistry. Its crystal structure was determined at 100 K. Both weak intra­molecular O—H⋯O and inter­molecular O—H⋯S hydrogen bonding can be observed.

## Chemical context   

Acyclic and cyclic di­thio­ether compounds containing the –S–C(*R*)(H)–S– (*R* = H, alkyl, ar­yl) motif are synthesized by nucleophilic substitution of geminal dihalides *X*–C(*R*)(H)–*X* in the presence of thiol­ate *R*S^−^ (Murray *et al.*, 1981 ▸). Alternatively, they are readily accessible by treatment of aldehydes and ketones with thiols *R*SH and di­thiols HS(CH_2_)_*n*_SH (*n* = 2, 3), yielding geminal di­thio ethers, also called acyclic and cyclic thio­acetals (1,3-di­thiol­anes, 1,3-di­thia­nes) (Shaterian *et al.*, 2011 ▸). This type of organosulfur compound is commonly used for Corey–Seebach umpolung reactions and the Mozingo reduction of di­thio­ketals to hydro­carbons (Seebach & Corey, 1975 ▸; Zhao *et al.*, 2017 ▸), but there are also numerous other transformations in organic chemistry such as their oxidation to sulfoxides and sulfones (Gasparrini *et al.*, 1984 ▸). They have also been used in the past as monodentate, chelating or bridging ligands to construct both simple mono- and dinuclear coordination compounds or to assemble coordination networks of varying dimensionality ranging from 1D to 3D. Selected examples are [(C_5_H_5_)Fe(CO)_2_(κ^1^-BzSCH_2_SBz)]^+^, the 1:1 adduct [Hg_2_(NO_3_)_2_·BzSCH_2_SBz)], the dinuclear Pd^I^ complex [ClPd(μ_2_-BzSCH_2_SBz)_2_PdCl], and the monodimensional coordination polymer [Ag_2_(BzSCH_2_SBz)_2_](ClO_4_)_2_ built upon dinuclear [Ag(μ_2_-BzSCH_2_SBz)_2_Ag]^2+^ units (Brodersen & Rölz, 1977 ▸; Fuchita *et al.*, 1991 ▸; Kuhn & Schumann, 1986 ▸; Li *et al.*, 2005 ▸).

In the context of our research inter­est in the assembly of mol­ecular cluster compounds and coordination polymers by complexation of ArSCH_2_SAr or di­thiol­ane- and di­thiane-based thia­heterocycles (Chaabéne *et al.*, 2016 ▸; Knauer *et al.*, 2020 ▸; Knorr *et al.*, 2014 ▸; Raghuvanshi *et al.*, 2017 ▸, 2019 ▸; Schlachter *et al.*, 2018 ▸) , we have developed novel functionalized di­thio ether compounds such as ferrocenyl thio­ethers bearing a substituent at the α-carbon atom linking the two –S*R* groups. With the idea of designing a functionalized thio­acetal ligand bearing additional harder O-donor sites along with the two soft S-donor sites, we chose 2-hy­droxy-3-meth­oxy­benzaldehyde (*ortho*-vanillin) as the starting material. This hy­droxy­lated aldehyde is present in the extracts and essential oils of many plants. Several papers describe also its use (in its deprotonated vanillinato form or as a Schiff base-derived ligand) in coordination chemistry (Andruh, 2015 ▸; Kırpık *et al.*, 2019 ▸; Yu *et al.*, 2011 ▸). Its reaction with 2 equivalents of benzyl mercaptan affords the targeted di­thio­acetal 2-hy­droxy-3-meth­oxy­phen­yl[bis­(benzyl­thio)]methane, **1**, which was isolated in high yield as a crystalline solid.

This acyclic thio­acetal contains, in addition to the benzylic thio ether groups and the meth­oxy group prone to ligate metal centres, a phenolic hydroxyl group, which may allow additional inter­actions through hydrogen bonding. 
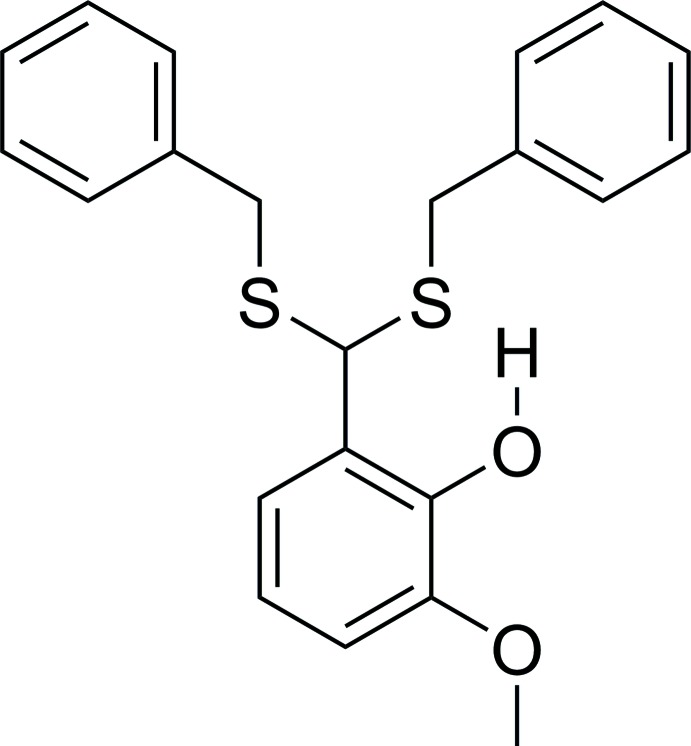


## Structural commentary   

**Figure 1:**
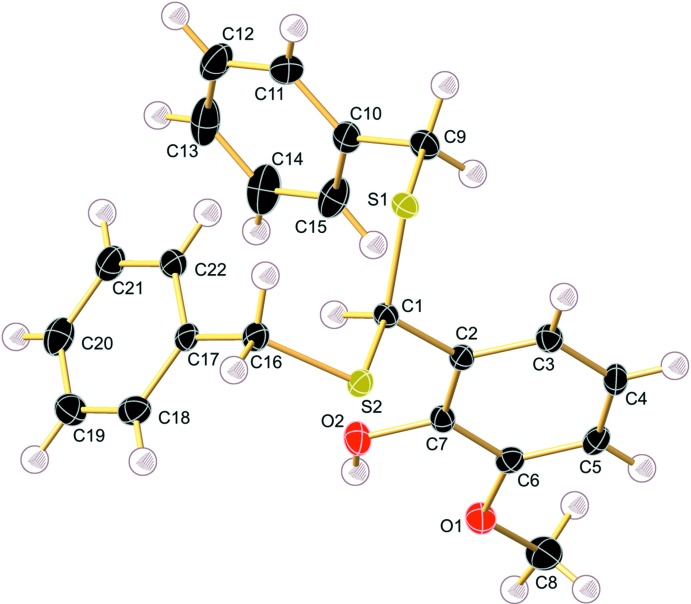
The mol­ecular structure of **1** with displacement ellipsoids drawn at the 50% probability level.

Compound **1** crystallizes from CH_2_Cl_2_/hexane in the ortho­rhom­bic crystal system, space group *Pbca*. The C1—S1 and C1—S2 bond lengths of 1.8132 (12) and 1.8189 (12) Å are comparable with those of [BzSC(H)(C_6_H_4_NO_2_-*p*)SBz] [1.823 (3) and 1.8262 (19) Å], but are elongated compared with those of bis­(benzyl­sulfan­yl)methane (CSD TUQPAX) [1.7988 (13) and 1.8013 (13) Å; Yang *et al.*, 2010 ▸). The angle S1—C1—S2 is almost identical with that of 4-nitro­phenyl-bis­(benzyl­sulfan­yl)methane [107.26 (6) *versus* 107.76°], but considerably more acute than in [BzSCH_2_SBz] [117.33 (7)°]. There is a weak intra­molecular O1⋯H2 contact of 2.17 (2) Å between the H atom of the phenolic hydroxyl group and the O-atom of the meth­oxy group (Table 1 ▸). For the starting material, 2-hy­droxy-3-meth­oxy­benzaldehyde, a similar intra­molecular hydrogen bond seems to be absent; instead, a rather strong intra­molecular hydrogen bond between the O—H group and the carbonyl oxygen was found (Iwasaki *et al.*, 1976 ▸). The phenyl rings of the benzyl groups (C10–C15) and (C17–C22) and the phenyl ring of the vanillin unit (C2–C7) form dihedral angles of 35.38 (6) and 79.77 (6)°, respectively. Compared to the structurally very closely related compound 4-nitro­phenyl-bis­(benzyl­sulfan­yl)methane [BzSC(H)(C_6_H_4_NO_2_-*p*)SBz] (SUNMAQ; Binkowska *et al.*, 2009 ▸), the coplanar and perpendicular arrangement of the phenyl rings is thus lost in **1** (Figs. 1 ▸ and 2 ▸).

## Supra­molecular features   

**Figure 3:**
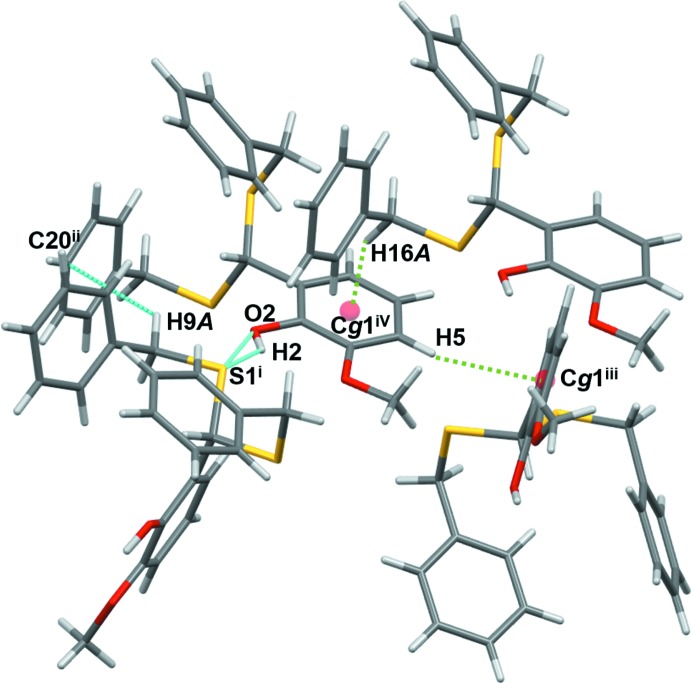
Inter­molecular contacts for compound **1**. Symmetry codes as in Table 1 ▸.

In the crystal, there is an O—H⋯S hydrogen bond between the H2 atom of the phenolic hydroxyl group and the S1 atom of a neighbouring mol­ecule with distances [H2⋯S1 = 2.44 (2), O2⋯S1 = 3.1315 (13) Å] similar to those reported for 4-(1,3-di­thian-2-yl)-1,2-benzene­diol [H⋯S = 2.44, O⋯S = 3.2417 (13) Å], while the O—H⋯S angle is more acute [139.0 (17) *versus* 159.2°] (Fig. 3 ▸ and Table 1 ▸). This O2—H2⋯S1 inter­action results in the formation of chains running along the *b*-axis direction.

The benzylic methyl­ene group on sulfur atom S2 inter­acts with the π-cloud of the phenyl part of the vanillin unit through a C—H⋯π inter­action (Table 1 ▸). The second phenyl ring of the di­thiane unit also exhibits a C—H⋯π inter­action: the second methyl­ene group on sulfur atom S1 inter­acts with a phenyl carbon. The third C—H⋯π contact is between adjacent vanillin units.

## Database survey   

There are several other examples of structurally characterized related di­thioethers bearing hy­droxy substituents that give rise to the formation of supra­molecular networks. Selected examples found in the Cambridge Structural Database (CSD, version 5.40, update August 2019; Groom *et al.*, 2016 ▸) include 2-(2-hy­droxy­phen­yl)-1,3-di­thiane (WADROJ; Usman *et al.*, 2003 ▸), 2-(3-hy­droxy­phen­yl)-1,3-di­thiane (KALJUD; Ganguly *et al.*, 2005 ▸), 4,6-bis­(1,3-di­thian-2-yl)benzene-1,3-diol (DITFIX; Datta *et al.*, 2013 ▸), 4-(1,3-di­thian-2-yl)benzene-1,3-diol (DITFOD; Datta *et al.*, 2013 ▸), 2-phenyl-1,3-dithiepane-5,6-diol (FIBTOC; Liu *et al.*, 2018 ▸) and 2,2′-{[(4-meth­oxy­phen­yl)methyl­ene]disulfanedi­yl}di­ethanol (YISVUT; Laskar *et al.*, 2013 ▸). It is noteable that in most of these examples, the inter­molecular contacts are noticeably stronger than those of **1**.

Note that in di­thio­ether compounds with phenolic aryl groups as encountered in **1**, the relative position of the phenolic OH substituent seems to play a crucial role, whether the inter­molecular contacts are dominated by O—H⋯H or O—H⋯S hydrogen bonds. This is nicely illustrated by the series of three isomeric hy­droxy­phenyl-1,3-di­thia­nes, *ortho*-, *meta*- and *para*-HO–C_6_H_4_–C_4_H_7_S_2_. Whereas 2-(2-hy­droxy­phen­yl)-1,3-di­thiane (WADROY) and 2-(3-hy­droxy­phen­yl)-1,3-di­thiane (KALJUD) exhibit, like **1**, only inter­molecular O—H⋯S hydrogen bonding, the *para*-derivative 2-(4-hy­droxy­phen­yl)-1,3-di­thiane (KALKAK) features solely inter­molecular phenolic O—H⋯H bonding (Ganguly *et al.*, 2005).

## Synthesis and crystallization   

**Figure 4:**
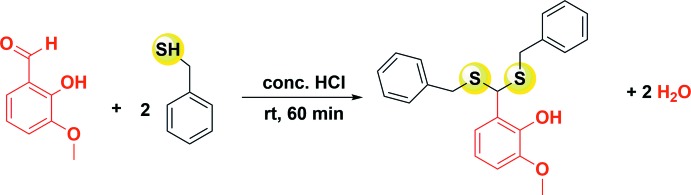
Synthesis of **1**.

The reaction scheme for the synthesis of the title compound is illustrated in Fig. 4 ▸.

3-Meth­oxy­salicyl­aldehyde (1 mmol, 152 mg), benzyl mercaptan (2.5 mmol, 310 mg), and conc. HCl (2 mL) were added to a flask at 273 K. The mixture was stirred for 60 min at room temperature. After the reaction was complete, the resulting mixture was neutralized with 10% aq NaHCO_3_ (10 mL) and extracted with di­chloro­methane (3 × 10 mL). The combined extracts were washed with H_2_O (3 × 20 mL) and dried over Na_2_SO_4_. Evaporation of the solvent *in vacuo* gave a solid product, which was further purified by column chromatography. The product was obtained as a white solid, Yield: 83% (430 mg). X-ray quality crystals were obtained by keeping a di­chloro­methane:hexane (1:1) mixture of **1** at 278 K for 3–4 d. ^1^H NMR (400 MHz, CDCl_3_) δ 7.26–7.19 (*m*, 11H, Ph), 6.86 (*t*, *J* = 7.8 Hz, 1H, CH), 6.78 (*d*, *J* = 7.8 Hz, 1H, CH), 5.83 (*s*, 1H, OH), 5.13 (*s*, 1H CHS_2_), 3.88 (*s*, 3H, OCH_3_), 3.79 (*d*, *J* = 13.1 Hz, 2H, CH_2_), 3.64 (*d*, *J* = 13.1 Hz, 2H, CH_2_).^13^C{^1^H} NMR (101 MHz, CDCl_3_) δ 146.5 (*C*q_OH_), 142.8 (*C*qOCH_3_), 137.8 (SCH_2_ *C*q), 129.1 (SCH_2_C*C*H), 128.4 (SCH_2_CCH*C*H), 126.9 (SCH_2_CCHCH*C*H), 125.3 (S_2_CH*C*q), 120.9 (S_2_CHCq*C*H), 119.9 (S_2_CHCqCH*C*H), 110.0 (*C*HCqOCH_3_), 56.1 (O*C*H_3_), 44.8 (S_2_ *C*H), 36.7 (S_2_ *C*H_2_). IR (ATR) cm^−1^: 3419 (O—H), 1430-1612 (C=C). 1054 and 1264 (C—O), 766 (C—S). HRMS: (ESI) *m*/*z* calculated for C_22_H_22_O_2_S_2_Na [*M* + Na]^+^ 405.0953, found 405.0965.

## Refinement   

Crystal data, data collection and structure refinement details are summarized in Table 2 ▸. H atoms were positioned geometrically (C—H = 0.95–1.00 Å) and refined using a riding model, with *U*_iso_(H) = 1.2*U*_eq_(C) for CH_2_ and CH hydrogen atoms and *U*_iso_(H) = 1.5*U*_eq_(C-meth­yl). The phenolic proton H2 was refined independently.

## Supplementary Material

Crystal structure: contains datablock(s) I. DOI: 10.1107/S2056989020002091/vm2228sup1.cif

Supporting information file. DOI: 10.1107/S2056989020002091/vm2228Isup3.cdx

Supporting information file. DOI: 10.1107/S2056989020002091/vm2228Isup3.cml

CCDC reference: 1983985

Additional supporting information:  crystallographic information; 3D view; checkCIF report

## Figures and Tables

**Figure 2:**
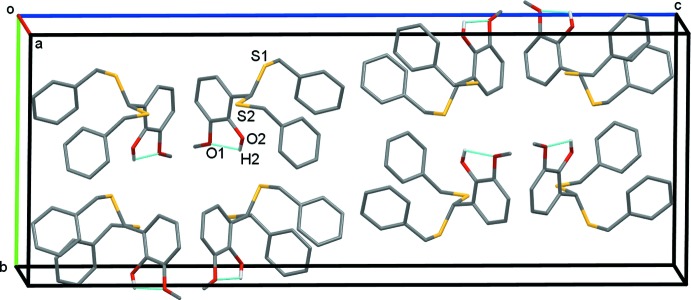
A view of the crystal packing of the title compound. For clarity, H atoms have been omitted. The intramolecular O1⋯H2 contacts are shown as dashed lines. For clarity, only H atoms involved in these interactions are presented. The intermolecular contacts are shown in Fig. 3 ▸.

**Table 1:** Hydrogen-bond geometry (Å, °) *Cg*1 is the centroid of the C2–C7 ring.

*D*—H⋯*A*	*D*—H	H⋯*A*	*D*⋯*A*	*D*—H⋯*A*
O2—H2⋯S1^i^	0.85 (2)	2.44 (2)	3.1315 (13)	139.0 (17)
O2—H2⋯O1	0.85 (2)	2.17 (2)	2.6469 (16)	115.4 (16)
C9—H9*A*⋯C20^ii^	0.99	2.86	3.528 (2)	125
C5—H5⋯*Cg*1^iii^	0.95	2.84	3.7487 (15)	160
C16—H16*A*⋯*Cg*1^iv^	0.99	2.71	3.6316 (15)	154

Symmetry codes: (i) 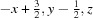; (ii) 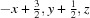; (iii) 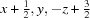; (iv) 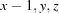.

**Table 2:** Experimental details

Crystal data	
Chemical formula	C_22_H_22_O_2_S_2_
*M*_r_	382.51
Crystal system, space group	Orthorhombic, *P* *b* *c* *a*
Temperature (K)	100
*a*, *b*, *c* (Å)	7.7418 (8), 13.856 (3), 36.197 (5)
*V* (Å^3^)	3882.9 (10)
*Z*	8
Radiation type	Mo *K*α
μ (mm^−1^)	0.29
Crystal size (mm)	0.49 × 0.42 × 0.25

Data collection	
Diffractometer	Bruker D8 VENTURE area detector
Absorption correction	Multi-scan (*SADABS*; Bruker, 2016 ▸)
*T*_min_, *T*_max_	0.713, 0.746
No. of measured, independent and observed [*I* > 2σ(*I*)] reflections	107706, 5005, 4510
*R*_int_	0.033
(sin θ/λ)_max_ (Å^−1^)	0.684

Refinement	
*R*[*F* ^2^ > 2σ(*F* ^2^)], *wR*(*F* ^2^), *S*	0.034, 0.079, 1.09
No. of reflections	5005
No. of parameters	241
H-atom treatment	H atoms treated by a mixture of independent and constrained refinement
Δρ_max_, Δρ_min_ (e Å^−3^)	0.34, −0.23

Computer programs: *APEX2* and *SAINT* (Bruker, 2016 ▸), *SHELXT* (Sheldrick, 2015*a* ▸), *SHELXL* (Sheldrick, 2015*b* ▸) and *OLEX2* (Dolomanov *et al.*, 2009 ▸).

## supplementary crystallographic information

### Crystal data

**Table:**

C_22_H_22_O_2_S_2_	*D*_x_ = 1.309 Mg m^−^^3^
*M**_r_* = 382.51	Mo *K*α radiation, λ = 0.71073 Å
Orthorhombic, *P**b**c**a*	Cell parameters from 9791 reflections
*a* = 7.7418 (8) Å	θ = 2.3–28.2°
*b* = 13.856 (3) Å	µ = 0.29 mm^−^^1^
*c* = 36.197 (5) Å	*T* = 100 K
*V* = 3882.9 (10) Å^3^	Block, colourless
*Z* = 8	0.49 × 0.42 × 0.25 mm
*F*(000) = 1616	

### Data collection

**Table:**

Bruker D8 VENTURE area detector diffractometer	5005 independent reflections
Radiation source: microfocus sealed X-ray tube, Incoatec Iµs	4510 reflections with *I* > 2σ(*I*)
HELIOS mirror optics monochromator	*R*_int_ = 0.033
Detector resolution: 10.4167 pixels mm^-1^	θ_max_ = 29.1°, θ_min_ = 2.3°
ω and φ scans	*h* = −9→10
Absorption correction: multi-scan (SADABS; Bruker, 2016)	*k* = −18→18
*T*_min_ = 0.713, *T*_max_ = 0.746	*l* = −49→45
107706 measured reflections	

### Refinement

**Table:**

Refinement on *F*^2^	Hydrogen site location: mixed
Least-squares matrix: full	H atoms treated by a mixture of independent and constrained refinement
*R*[*F*^2^ > 2σ(*F*^2^)] = 0.034	*w* = 1/[σ^2^(*F*_o_^2^) + (0.0287*P*)^2^ + 2.6399*P*] where *P* = (*F*_o_^2^ + 2*F*_c_^2^)/3
*wR*(*F*^2^) = 0.079	(Δ/σ)_max_ = 0.003
*S* = 1.09	Δρ_max_ = 0.34 e Å^−^^3^
5005 reflections	Δρ_min_ = −0.23 e Å^−^^3^
241 parameters	Extinction correction: SHELXL-2018/3 (Sheldrick 2015b), Fc^*^=kFc[1+0.001xFc^2^λ^3^/sin(2θ)]^-1/4^
0 restraints	Extinction coefficient: 0.0017 (2)
Primary atom site location: dual	

### Special details

**Table:**

Geometry. All esds (except the esd in the dihedral angle between two l.s. planes) are estimated using the full covariance matrix. The cell esds are taken into account individually in the estimation of esds in distances, angles and torsion angles; correlations between esds in cell parameters are only used when they are defined by crystal symmetry. An approximate (isotropic) treatment of cell esds is used for estimating esds involving l.s. planes.

### Fractional atomic coordinates and isotropic or equivalent isotropic displacement parameters (Å^2^)

**Table:**

	*x*	*y*	*z*	*U*_iso_*/*U*_eq_	
S1	0.61115 (4)	0.82824 (2)	0.63211 (2)	0.01667 (8)	
S2	0.42202 (4)	0.68918 (2)	0.67748 (2)	0.01772 (8)	
O1	1.02466 (13)	0.48649 (7)	0.70693 (3)	0.0234 (2)	
O2	0.76523 (13)	0.52864 (7)	0.66212 (3)	0.0228 (2)	
H2	0.826 (3)	0.4778 (15)	0.6649 (5)	0.043 (5)*	
C1	0.62315 (15)	0.70907 (8)	0.65265 (3)	0.0146 (2)	
H1	0.631957	0.660278	0.632378	0.017*	
C2	0.77100 (15)	0.69329 (8)	0.67929 (3)	0.0147 (2)	
C3	0.84314 (17)	0.76632 (9)	0.70092 (3)	0.0179 (2)	
H3	0.799141	0.830165	0.699171	0.022*	
C4	0.97814 (17)	0.74678 (10)	0.72490 (3)	0.0202 (3)	
H4	1.025853	0.797416	0.739354	0.024*	
C5	1.04472 (16)	0.65382 (9)	0.72807 (3)	0.0185 (2)	
H5	1.137719	0.640764	0.744446	0.022*	
C6	0.97346 (16)	0.58102 (9)	0.70704 (3)	0.0172 (2)	
C7	0.83624 (16)	0.60032 (9)	0.68273 (3)	0.0157 (2)	
C8	1.17946 (19)	0.46331 (11)	0.72664 (5)	0.0311 (3)	
H8A	1.274327	0.503799	0.717632	0.047*	
H8B	1.208119	0.395191	0.722705	0.047*	
H8C	1.161929	0.475073	0.753070	0.047*	
C9	0.81405 (17)	0.83571 (10)	0.60670 (4)	0.0222 (3)	
H9A	0.828248	0.902523	0.597491	0.027*	
H9B	0.910248	0.822557	0.623999	0.027*	
C10	0.82841 (17)	0.76755 (10)	0.57454 (4)	0.0216 (3)	
C11	0.7673 (2)	0.79298 (12)	0.53965 (4)	0.0294 (3)	
H11	0.712651	0.853694	0.536144	0.035*	
C12	0.7855 (2)	0.73040 (14)	0.51001 (4)	0.0354 (4)	
H12	0.742688	0.748223	0.486385	0.043*	
C13	0.8656 (2)	0.64242 (14)	0.51472 (4)	0.0367 (4)	
H13	0.880248	0.600256	0.494264	0.044*	
C14	0.9247 (2)	0.61563 (13)	0.54931 (5)	0.0360 (4)	
H14	0.978548	0.554677	0.552712	0.043*	
C15	0.90510 (18)	0.67790 (11)	0.57899 (4)	0.0275 (3)	
H15	0.944821	0.658858	0.602728	0.033*	
C16	0.26715 (16)	0.68531 (9)	0.63950 (3)	0.0188 (2)	
H16A	0.150774	0.672722	0.649760	0.023*	
H16B	0.264227	0.749436	0.627453	0.023*	
C17	0.30595 (16)	0.61034 (9)	0.61075 (3)	0.0176 (2)	
C18	0.25087 (18)	0.51529 (10)	0.61502 (4)	0.0231 (3)	
H18	0.188646	0.497061	0.636537	0.028*	
C19	0.2863 (2)	0.44732 (10)	0.58807 (4)	0.0274 (3)	
H19	0.246189	0.382978	0.590983	0.033*	
C20	0.37995 (19)	0.47228 (11)	0.55688 (4)	0.0264 (3)	
H20	0.406356	0.424957	0.538749	0.032*	
C21	0.43459 (18)	0.56639 (12)	0.55233 (4)	0.0269 (3)	
H21	0.498143	0.584070	0.530921	0.032*	
C22	0.39681 (17)	0.63509 (10)	0.57896 (4)	0.0223 (3)	
H22	0.433413	0.699893	0.575465	0.027*	

### Atomic displacement parameters (Å^2^)

**Table:**

	*U*^11^	*U*^22^	*U*^33^	*U*^12^	*U*^13^	*U*^23^
S1	0.01657 (15)	0.01393 (14)	0.01952 (15)	−0.00028 (11)	−0.00002 (11)	0.00077 (10)
S2	0.01469 (15)	0.02377 (16)	0.01471 (14)	−0.00302 (11)	−0.00020 (11)	0.00089 (11)
O1	0.0226 (5)	0.0196 (4)	0.0279 (5)	0.0043 (4)	−0.0075 (4)	0.0010 (4)
O2	0.0254 (5)	0.0145 (4)	0.0285 (5)	0.0022 (4)	−0.0092 (4)	−0.0048 (4)
C1	0.0133 (5)	0.0145 (5)	0.0159 (5)	−0.0009 (4)	−0.0003 (4)	0.0000 (4)
C2	0.0129 (5)	0.0164 (5)	0.0147 (5)	−0.0011 (4)	0.0002 (4)	−0.0002 (4)
C3	0.0184 (6)	0.0174 (6)	0.0180 (6)	−0.0009 (5)	−0.0005 (5)	−0.0022 (4)
C4	0.0207 (6)	0.0231 (6)	0.0169 (6)	−0.0049 (5)	−0.0014 (5)	−0.0035 (5)
C5	0.0147 (6)	0.0266 (6)	0.0141 (5)	−0.0014 (5)	−0.0013 (4)	0.0017 (5)
C6	0.0166 (6)	0.0193 (6)	0.0158 (5)	0.0013 (5)	0.0007 (4)	0.0022 (4)
C7	0.0146 (6)	0.0172 (5)	0.0154 (5)	−0.0018 (4)	0.0003 (4)	−0.0012 (4)
C8	0.0235 (7)	0.0270 (7)	0.0428 (9)	0.0027 (6)	−0.0105 (6)	0.0110 (6)
C9	0.0189 (6)	0.0261 (6)	0.0217 (6)	−0.0069 (5)	0.0026 (5)	0.0003 (5)
C10	0.0148 (6)	0.0315 (7)	0.0186 (6)	−0.0065 (5)	0.0025 (5)	−0.0010 (5)
C11	0.0307 (8)	0.0356 (8)	0.0221 (6)	−0.0084 (6)	−0.0008 (6)	0.0060 (6)
C12	0.0323 (8)	0.0572 (10)	0.0168 (6)	−0.0122 (8)	−0.0003 (6)	0.0013 (6)
C13	0.0223 (7)	0.0615 (11)	0.0264 (7)	−0.0047 (7)	0.0033 (6)	−0.0175 (7)
C14	0.0210 (7)	0.0494 (10)	0.0376 (8)	0.0090 (7)	−0.0038 (6)	−0.0153 (7)
C15	0.0167 (6)	0.0423 (8)	0.0236 (7)	0.0046 (6)	−0.0036 (5)	−0.0051 (6)
C16	0.0131 (6)	0.0258 (6)	0.0175 (6)	−0.0020 (5)	−0.0024 (5)	0.0022 (5)
C17	0.0121 (5)	0.0250 (6)	0.0157 (5)	−0.0019 (5)	−0.0037 (4)	0.0027 (5)
C18	0.0216 (6)	0.0254 (6)	0.0222 (6)	−0.0030 (5)	−0.0006 (5)	0.0061 (5)
C19	0.0304 (8)	0.0213 (6)	0.0306 (7)	−0.0009 (6)	−0.0054 (6)	0.0034 (5)
C20	0.0227 (7)	0.0319 (7)	0.0247 (7)	0.0049 (6)	−0.0067 (5)	−0.0060 (6)
C21	0.0219 (7)	0.0411 (8)	0.0177 (6)	−0.0061 (6)	0.0007 (5)	−0.0017 (6)
C22	0.0205 (6)	0.0282 (7)	0.0182 (6)	−0.0083 (5)	−0.0017 (5)	0.0022 (5)

### Geometric parameters (Å, º)

**Table:**

S1—C1	1.8132 (12)	C10—C11	1.3940 (19)
S1—C9	1.8232 (14)	C10—C15	1.386 (2)
S2—C1	1.8189 (12)	C11—H11	0.9500
S2—C16	1.8250 (13)	C11—C12	1.387 (2)
O1—C6	1.3685 (15)	C12—H12	0.9500
O1—C8	1.4312 (17)	C12—C13	1.378 (3)
O2—H2	0.85 (2)	C13—H13	0.9500
O2—C7	1.3584 (15)	C13—C14	1.383 (2)
C1—H1	1.0000	C14—H14	0.9500
C1—C2	1.5126 (16)	C14—C15	1.386 (2)
C2—C3	1.3960 (17)	C15—H15	0.9500
C2—C7	1.3893 (17)	C16—H16A	0.9900
C3—H3	0.9500	C16—H16B	0.9900
C3—C4	1.3854 (18)	C16—C17	1.5007 (18)
C4—H4	0.9500	C17—C18	1.3929 (18)
C4—C5	1.3920 (19)	C17—C22	1.3917 (17)
C5—H5	0.9500	C18—H18	0.9500
C5—C6	1.3789 (18)	C18—C19	1.383 (2)
C6—C7	1.4051 (17)	C19—H19	0.9500
C8—H8A	0.9800	C19—C20	1.385 (2)
C8—H8B	0.9800	C20—H20	0.9500
C8—H8C	0.9800	C20—C21	1.381 (2)
C9—H9A	0.9900	C21—H21	0.9500
C9—H9B	0.9900	C21—C22	1.386 (2)
C9—C10	1.5031 (19)	C22—H22	0.9500
			
C1—S1—C9	102.38 (6)	C15—C10—C9	120.31 (12)
C1—S2—C16	101.22 (6)	C15—C10—C11	118.51 (13)
C6—O1—C8	117.12 (11)	C10—C11—H11	119.7
C7—O2—H2	108.5 (14)	C12—C11—C10	120.54 (15)
S1—C1—S2	107.26 (6)	C12—C11—H11	119.7
S1—C1—H1	108.6	C11—C12—H12	119.9
S2—C1—H1	108.6	C13—C12—C11	120.22 (14)
C2—C1—S1	115.59 (8)	C13—C12—H12	119.9
C2—C1—S2	108.11 (8)	C12—C13—H13	120.1
C2—C1—H1	108.6	C12—C13—C14	119.86 (15)
C3—C2—C1	123.74 (11)	C14—C13—H13	120.1
C7—C2—C1	117.80 (10)	C13—C14—H14	120.1
C7—C2—C3	118.45 (11)	C13—C14—C15	119.88 (16)
C2—C3—H3	119.6	C15—C14—H14	120.1
C4—C3—C2	120.77 (12)	C10—C15—H15	119.5
C4—C3—H3	119.6	C14—C15—C10	120.96 (14)
C3—C4—H4	119.6	C14—C15—H15	119.5
C3—C4—C5	120.79 (12)	S2—C16—H16A	108.7
C5—C4—H4	119.6	S2—C16—H16B	108.7
C4—C5—H5	120.6	H16A—C16—H16B	107.6
C6—C5—C4	118.90 (12)	C17—C16—S2	114.28 (9)
C6—C5—H5	120.6	C17—C16—H16A	108.7
O1—C6—C5	125.87 (12)	C17—C16—H16B	108.7
O1—C6—C7	113.53 (11)	C18—C17—C16	121.08 (11)
C5—C6—C7	120.59 (12)	C22—C17—C16	120.27 (12)
O2—C7—C2	118.79 (11)	C22—C17—C18	118.65 (12)
O2—C7—C6	120.71 (11)	C17—C18—H18	119.8
C2—C7—C6	120.50 (11)	C19—C18—C17	120.31 (13)
O1—C8—H8A	109.5	C19—C18—H18	119.8
O1—C8—H8B	109.5	C18—C19—H19	119.7
O1—C8—H8C	109.5	C18—C19—C20	120.56 (13)
H8A—C8—H8B	109.5	C20—C19—H19	119.7
H8A—C8—H8C	109.5	C19—C20—H20	120.2
H8B—C8—H8C	109.5	C21—C20—C19	119.55 (13)
S1—C9—H9A	108.6	C21—C20—H20	120.2
S1—C9—H9B	108.6	C20—C21—H21	120.0
H9A—C9—H9B	107.6	C20—C21—C22	120.06 (13)
C10—C9—S1	114.75 (9)	C22—C21—H21	120.0
C10—C9—H9A	108.6	C17—C22—H22	119.6
C10—C9—H9B	108.6	C21—C22—C17	120.83 (13)
C11—C10—C9	121.18 (13)	C21—C22—H22	119.6
			
S1—C1—C2—C3	30.00 (15)	C7—C2—C3—C4	0.84 (18)
S1—C1—C2—C7	−151.09 (9)	C8—O1—C6—C5	−8.60 (19)
S1—C9—C10—C11	87.20 (15)	C8—O1—C6—C7	170.97 (12)
S1—C9—C10—C15	−93.52 (14)	C9—S1—C1—S2	−177.44 (6)
S2—C1—C2—C3	−90.18 (13)	C9—S1—C1—C2	61.92 (10)
S2—C1—C2—C7	88.73 (12)	C9—C10—C11—C12	178.27 (13)
S2—C16—C17—C18	85.08 (14)	C9—C10—C15—C14	−177.74 (14)
S2—C16—C17—C22	−95.56 (13)	C10—C11—C12—C13	−0.4 (2)
O1—C6—C7—O2	0.73 (17)	C11—C10—C15—C14	1.6 (2)
O1—C6—C7—C2	−179.12 (11)	C11—C12—C13—C14	1.4 (2)
C1—S1—C9—C10	66.89 (11)	C12—C13—C14—C15	−0.8 (2)
C1—S2—C16—C17	56.83 (10)	C13—C14—C15—C10	−0.6 (2)
C1—C2—C3—C4	179.74 (11)	C15—C10—C11—C12	−1.0 (2)
C1—C2—C7—O2	0.19 (17)	C16—S2—C1—S1	66.50 (7)
C1—C2—C7—C6	−179.95 (11)	C16—S2—C1—C2	−168.23 (8)
C2—C3—C4—C5	−0.18 (19)	C16—C17—C18—C19	179.38 (12)
C3—C2—C7—O2	179.17 (11)	C16—C17—C22—C21	179.52 (12)
C3—C2—C7—C6	−0.98 (18)	C17—C18—C19—C20	1.3 (2)
C3—C4—C5—C6	−0.34 (19)	C18—C17—C22—C21	−1.1 (2)
C4—C5—C6—O1	179.74 (12)	C18—C19—C20—C21	−1.5 (2)
C4—C5—C6—C7	0.19 (19)	C19—C20—C21—C22	0.4 (2)
C5—C6—C7—O2	−179.67 (11)	C20—C21—C22—C17	0.9 (2)
C5—C6—C7—C2	0.48 (19)	C22—C17—C18—C19	0.0 (2)

### Hydrogen-bond geometry (Å, º)

*Cg*1 is the centroid of the C2–C7 ring.

**Table:**

*D*—H···*A*	*D*—H	H···*A*	*D*···*A*	*D*—H···*A*
O2—H2···S1^i^	0.85 (2)	2.44 (2)	3.1315 (13)	139.0 (17)
O2—H2···O1	0.85 (2)	2.17 (2)	2.6469 (16)	115.4 (16)
C9—H9*A*···C20^ii^	0.99	2.86	3.528 (2)	125
C5—H5···*Cg*1^iii^	0.95	2.84	3.7487 (15)	160
C16—H16*A*···*Cg*1^iv^	0.99	2.71	3.6316 (15)	154

Symmetry codes: (i) −*x*+3/2, *y*−1/2, *z*; (ii) −*x*+3/2, *y*+1/2, *z*; (iii) *x*+1/2, *y*, −*z*+3/2; (iv) *x*−1, *y*, *z*.
